# Palliative care needs among patients with advanced illnesses in Bhutan

**DOI:** 10.1186/s12904-020-00697-9

**Published:** 2021-01-09

**Authors:** Tara Devi Laabar, Christobel Saunders, Kirsten Auret, Claire E. Johnson

**Affiliations:** 1 Medical School, The University of Western Australia, 35 Stirling Highway, 6009 Perth, Western Australia Australia; 2 Department of Nursing, Faculty of Nursing and Public Health, Khesar Gyalpo University of Medical Sciences of Bhutan, Thimphu, Bhutan; 3 Medical School, Surgery Division, The University of Western Australia, 35 Stirling Highway, 6009 Perth, Western Australia Australia; 4 Rural Clinical School of Western Australia, The University of Western Australia, Science Building M701, 35 Stirling Terrace, 6330 Albany, Western Australia Australia; 5 Monash Nursing and Midwifery, Monash University, 10 Chancellors Walk, Wellington Road, 3800 Clayton, Victoria Australia; 6 Australian Health Services Research Institute (AHSRI), University of Wollongong, Building 234, Innovation Campus, 2522 Sydney, NSW Australia

**Keywords:** Bhutan, Palliative care, Advanced illnesses, Public health strategy, Symptoms, Function, cancer, Non‐malignant

## Abstract

**Background:**

Palliative care improves the quality of lives of patients and families affected by advanced illnesses through the prevention and relief of suffering. While palliative care is well established in developed countries, it is inadequate or non-existent in most developing countries. Palliative care is an emerging concept in Bhutan, a tiny Himalayan Kingdom. A small community palliative care service is available in the national referral hospital with three dedicated inpatient palliative care beds. This study explored the needs for palliative care among patients diagnosed with advanced illnesses and is a component of a larger project aimed to inform a suitable palliative care model for the country.

**Methods:**

This is a cross-sectional descriptive study. A survey, using a structured questionnaire including the EORTC QLQ-C30, was carried out among patients with advanced illness in hospitals, primary care units and communities across the country. Purposeful and snowball sampling strategies were used to recruit study participants.

**Results:**

Seventy (76%), out of 93 eligible patients, agreed to participate in the survey. Participants reported low to moderate scores on physical, role, emotional, cognitive and social functioning, a moderate score for the global health/ quality of life scale and moderately high (worse) scores in symptoms including fatigue, pain, insomnia, loss of appetite and the financial impact from the disease.

**Conclusions:**

The symptom burden experienced by patients affected by advanced illnesses demonstrates the need for palliative care in Bhutan. These findings will help inform the development of a public health-focused palliative care model, modified to the Bhutanese context, as recommended by the World Health Organization.

## Background

Advanced illnesses such as cancer and other non-malignant conditions can cause overwhelming suffering in patients and families demanding not only medical expertise but also support for the psychological, social, emotional, and spiritual distress throughout the disease trajectory [1]. Palliative care (PC), identified as a fundamental human right [2–4], is an approach to care that improves the quality of life of patients and families through the prevention and relief of suffering [5]. Provided across health care settings, PC is patient- and family-focussed, based on ethical principles, shared decision making, advanced care planning and excellent symptom management [6]. As a multidisciplinary approach, PC can be initiated at the diagnosis of a life-threatening disease along with the therapeutic management, continued throughout the disease trajectory until end-of-life and extended to grief and bereavement support of family members [7].

In response to continuous efforts by the World Health Organization (WHO) and other international organizations to ensure universal access [8, 9], PC services are mostly well established in developed countries [1, 10]. However, out of 56 million annual global deaths, almost 40 million happen in developing countries [11], and more than 33 million of those could benefit from PC [12]. In 2014, it was estimated that 78% of adults and 98% of children requiring PC were in low and middle-income-countries (LMIC) [1]. It is predicted that by 2060, 48 million people will die annually while experiencing serious health-related suffering and 83% of these deaths will occur in LMICs [13]. In 2014, the World Health Assembly declared that PC is an ethical responsibility of health systems and an ethical duty of all health care providers [14]. Tragically, PC still remains very minimal or non-existent in most developing countries [1, 10, 15–17]. The WHO, having identified several barriers in the provision of PC in developing countries, recognised PC as a public health priority and recommended four key strategies; (1) appropriate policies, (2) adequate availability of medications (including opioids), (3) education of health care workers and the public, and (4) implementation of PC services at all levels of health care [18, 19].

Bhutan, a tiny Himalayan Kingdom, a size similar to that of Switzerland, with a population of 771,608 [20], is landlocked between two giant nations of the world, China and India. Popularly known to the world for its concept of Gross National Happiness, Bhutan has made steady progress in modernization and poverty reduction since the early 1960s. Its annual per capita income stands at US$ 3438.16 [20], and the United Nations has recommended its eligibility to graduate to a middle income country by 2021 [21].

Healthcare in Bhutan is provided through a three-tiered system where the Basic Health Unit (BHU) functions at the primary level, district hospitals at the secondary level and the regional and national referral hospitals at the tertiary level. The integration of traditional medicine with the national health system is unique to Bhutan [22]. Having advanced both economically and socially, the disease patterns have altered significantly over the years. While vaccine-preventable diseases like polio are almost eradicated, infectious diseases such as HIV, dengue fever and Multi-Drug Resistant Tuberculosis (MDR-TB) are still on the rise [23]. Life expectancy has doubled from 35 in the 1960s to 70 in 2015 [24] and the incidence of non-communicable diseases like heart, lung, liver and kidney diseases and cancer are increasing [25]. Cancer is often diagnosed at an advanced stage in Bhutan [26].

Despite the growing number of people dying with conditions that are likely to benefit from PC, its development remains at a nascent stage in Bhutan. In 2018, a home-based PC group, consisting of a few nurses and doctors who received PC training in India, was initiated at the Jigme Dorji Wangchuck National Referral Hospital (JDWNRH) primarily for pain management for cancer patients. In addition, three beds were allocated for PC in the oncology ward in JDWNRH [26]. However, the needs for PC among patients with advanced illnesses have not been studied. This paper is a component of a larger study aimed to inform the development of a suitable PC model, socially, culturally and spiritually applicable, for Bhutan. The objective of this paper is to explore PC needs among patients with advanced illness.

## Methods

### Study design and setting

**Fig. 1:**
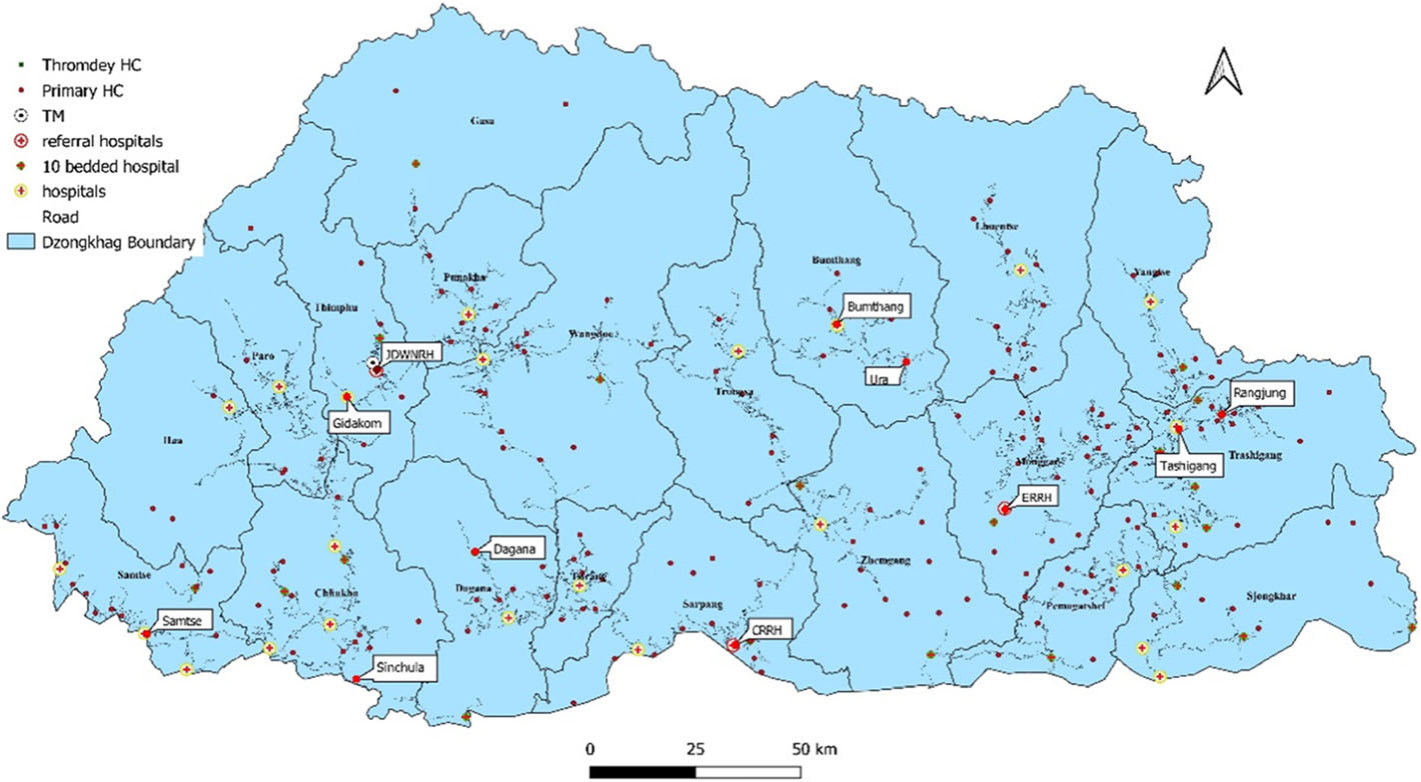
Map of Bhutan showing the study sites marked within the boxes. (Used with permission from the Policy & Planning Division, Ministry of Health, Bhutan

This study is a cross-sectional descriptive study using structured patient interviews. Data collection was scheduled for May to July 2019 in an attempt to avoid the usual monsoon season in Bhutan, which routinely disrupts travelling through the mountainous areas. Study sites included the JDWNRH, two regional referral hospitals, district hospitals and Basic Health Units (BHUs), both Grade I and Grade II, spread across Bhutan as indicated in Fig. 1. Basic Health Unit Grade I is a 10-bed community hospital equipped with basic diagnostic facilities managed by one or two general doctors along with a few nurses and allied health workers. Basic Health Unit Grade II is a primary health care centre managed by health assistants.

### Study population and sampling

Patients with advanced illness, defined in this study as having little chance of cure (e.g. cancer, heart, lung, kidney, liver failure, motor neuron disease, Parkinson’s disease, Huntington’s disease, Alzheimer’s disease/dementia, and HIV/ AIDS), and those at the end-of-life, in their last weeks/days of life - literally dying irrespective of the diagnosis, were included. The doctors, nurses and allied health care workers (particularly physiotherapists) helped in identifying the patients. We also recruited a gangrenous perineal wound which was likely to be ultimately incurable. Purposeful sampling, guided by diagnosis, end-of-life status, and willingness to participate, was used to recruit patients in the hospital. In the communities, using a snowball sampling strategy, patients nominated by clinicians were approached at their homes. Participants were excluded if they were < 18 years, diagnosed with acute illness, unconscious, semiconscious or delirious who could not give consent and those who were unwilling to participate. The final study size was determined pragmatically, in consideration of the eligible/willing participants and the duration of field work.

### Survey instrument

Data were collected using structured interviews to complete a questionnaire which included questions about socio-demographic characteristics, the clinical information of the patients and the European Organisation for Research and Treatment of Cancer (EORTC) quality of life questionnaire (EORTC QLQ-C30), see supplementary file. The latter is an integrated, 30-item questionnaire, to assess the health related quality of life (QOL) of cancer patients participating in both clinical trials as well as non-trial studies for which reliability and validity have already been established [27].

The EORTC QLQ-C30, used to determine the physical, psychological, social functioning and overall quality of life of patients, includes five functional scales (physical, role, emotional, cognitive, and social functions), three symptom scales (fatigue, nausea and vomiting, and pain), and a two-item global health and quality-of-life scale. The remaining six single items assess additional symptoms including dyspnoea, appetite loss, sleep disturbance, constipation, and diarrhoea, as well as the perceived financial impact of the disease and treatment. For the functional scales and global health status/QOL, the scores range from 0 to 100, with a higher score representing a higher level of functioning and QOL. The symptom scale scores also range from 0 to 100, however, higher scores represents a greater degree of symptoms or problems.

Although the EORTC QLQ-30 was specifically developed for cancer patients [27, 28], past studies [29] have used it to compare the QOL between cancer and non-cancer patients. Given the complexities of low literacy levels and prevalence of non-written dialects and, moreover, not every patient could read, write or speak the national language, translating the EORTC QLQ-C30 to *Dzongkha*, the national language, was not feasible. Thus, the EORTC QLQ-C30, English version, was considered to be the most appropriate tool for the study. The required consent to use this tool was obtained from the EORTC. A pilot test, conducted with four patients at JDWNRH, the two regional referral hospitals and Trashigang district hospital, found that the survey did not require changes as there were no issues regarding clarity and sensitivity of the questions.

### Data collection

Field notes were maintained to record any difficulties during data collection to allow TDL reflective consideration or potential bias during interpretation. To ensure that all potential participants were supported to be involved, generous time was set aside so that each patient was comfortable. Patients who could read and write English completed the survey questionnaire by themselves, if they wished. For the majority who could not read and write, TDL, who is fluent in all the main dialects used in Bhutan, was available to translate all information and the questionnaire at the bedside and completed the questionnaire in English on the participants’ behalf.

### Data analyses

Statistical analyses were conducted using the Statistical Analysis System (SAS) software, version 9.4 [30]. Firstly, descriptive statistics were calculated; means and standard deviation (SD) for interval data, and frequency and percentage for categorical data. There was no missing data. An independent sample T-test was conducted to compare the EORTC QLQ-30 scores between cancer and non-cancer patients. Statistical significance level was set at alpha 0.05. The graphical illustrations were generated using Microsoft Excel, version 16.0.

### Ethical consideration

Ethical approval was provided by the Human Research Ethics Committee at the University of Western Australia and the Research Ethics Board of Health in Bhutan with Reference numbers, RA/4/20/4990 and REBH/Approval/2018/097, respectively. While the overall administrative clearance for the study was provided by the Policy and Planning Division in the Ministry of Health in Bhutan, permission to access the patients was obtained from the administration of the individual study site. Informed consent was taken from every participant in the form of signature or thumb print.

## Results

### Field notes

Data collection was hampered by several unforeseen difficulties including a severe cyclone [31] which made travelling from one study site to another impossible, and a tragic accident [32] involving a public bus hit by a landslide, resulted in a government caution against public travel. Recruitment was slower than expected because a large number of potential participants were too ill, in pain or in distress to be approached. Most of the patients were illiterate, however, even many of those who could read and write preferred the researcher to read and explain the participant information form and felt most comfortable to fill the questionnaire along with the researcher. Only nine patients filled the questionnaire independently. None of the patients became distressed requiring assistance while completing the survey.

### Participants

Out of 93 eligible patients identified, 70 (76%) agreed to participate. Ten (14%) patients were recruited from the community and the remainder (60, 86%) from hospitals. Among the 23 patients who did not participate, 9 (39%) were too ill or in too much pain and distress and 8 (35%) were not able to provide consent due to being unconscious, semi-conscious, delirious or with hearing and speaking disability. Four (17%) patients were not allowed to participate by their family members who feared that their loved ones would be aware of the serious prognosis of the illness. The remaining 2 (9%) who declined to participate did not provide a specific reason. Eighty seven percent of the patients had to be guided through the questionnaire.

### Sociodemographic characteristics

Participating patients’ ages ranged from 18 to 85 years (mean 46.1, SD 15.5). The majority were married (53, 76%) and had at least 1 child (57, 81%). Among those who had more than 1 child, the eldest child’s age ranged from 6 to 69 years, (mean 25.6, SD 13.2) and the youngest child’s age from 1 to 47 years, (mean 16.8, SD 10.3). The sociodemographic characteristics are presented in Table 1.

**Table 1:** Sociodemographic characteristics of patients

	**Patients (n = 70)**	
	*n*	%
***Gender***		
Female	39	55.7
Male	31	44.3
***Marital status***		
Married	53	75.7
Never married	9	12.9
Widowed	4	5.7
Divorced	3	4.3
Separated	1	1.4
***Have Children***		
Yes	57	81.4
No	13	18.6
***How many children (n=57)***		
1 – 3	39	68.4
4 – 6	16	28.1
7 – 9	2	3.5
***Spoken language***		
* Sharchopkha*	28	40.0
* Dzongkha*	24	34.3
* Lhotshamkha*	12	17.1
Others^a^	6	8.6
***Education level***		
Never been to school	29	41.4
Higher secondary	10	14.3
Primary	9	12.9
Bachelors & above	8	11.4
Middle Secondary	6	8.6
Monastic/religious	5	7.1
Non-formal education	3	4.3
***Occupation***		
Housewife	24	34.3
Private/Corporate	15	21.4
Civil servant	8	11.4
Self-employed	4	5.7
Farmer	3	4.3
Others^b^	16	22.9

^a^*Bumthapkha, Khengkha, Kurtoepkha; *^b^Monk, nun, student, retired army, retired civil servant, not employed, security guard, NGO employee

### Clinical information of the patients

**Fig. 2:**
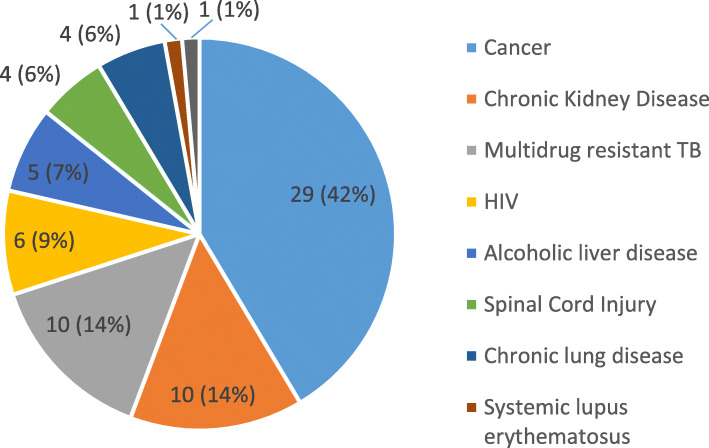
Diagnosis of the patients (*n=70*)

Sixty two (89%) patients knew their diagnosis and the remaining eight (11%) wished to know. A large minority of the patients (n = 29, 42%) were diagnosed with advanced cancer and the rest had a range of advanced non-malignant conditions (Fig. 2). The majority of the patients, (n = 46, 66%) reported that they were admitted to the hospital for 1 to 3 times, 16% for 4 to 6 times, and 10% for more than 7 times since diagnosis. For 41 (59%) patients, the longest admission to hospital lasted more than 2 weeks.

### Descriptive statistics for patients using EORTC-QLQ-30

The functional scales demonstrated low to moderate levels of functioning, with scores ranging from 32.1 for Role to 57.6 for the Cognitive Scale. The symptom scales including the single item symptoms ranged from low (good) with diarrhoea (20.0) through to moderate problems (fatigue 61.6) and a severe (worse) problems related to the financial impact of the illness (74.3). The mean score for the global health/QOL scale was 47.4. There were no differences in EORTC symptom or functioning subscales between cancer and non-cancer patients (*p* = < 0.05) except for constipation (*p* < 0.001). The results of the EORTC subscales are shown in Table 2.

**Table 2:** The EORTC QLQ-C30 mean scores and comparison between cancer and non-cancer patients

	Item^a^	Cancer patients		Non-cancer patients		Combined patients		*p*-Value (<0.05)
		Mean Score	(SD)	Mean Score	(SD)	Mean Score	(SD)	
Functioning scales^b^								
Physical	15-19	39.1	(31.4)	43.6	(34.6)	41.7	(33.1)	0.573
Role	20,21	31.6	(39.9)	32.5	(39.4)	32.1	(39.4)	0.925
Emotional	35-38	39.9	(38.0)	39.2	(37.6)	39.5	(37.5)	0.938
Cognitive	34,39	58.6	(27.7)	56.9	(27.4)	57.6	(27.3)	0.799
Social	40,41	43.7	(38.7)	33.7	(36.6)	37.9	(37.5)	0.283
Global health/ QOL scale^b^	43,44	48.6	(21.4)	46.5	(26.2)	47.4	(24.1)	0.724
Symptom scales and/or items^c^								
Fatigue	24,26,32	59.4	(34.8)	63.1	(29.8)	61.6	(31.7)	0.639
Nausea and vomiting	28,29	19.5	(30.6)	26.8	(33.5)	23.8	(32.3)	0.349
Pain	23,33	55.2	(35.7)	39.8	(37.2)	46.2	(37.1)	0.087
Dyspnoea	22	25.3	(34.1)	34.1	(37.6)	30.5	(36.2)	0.309
Insomnia	25	43.7	(39.9)	36.6	(37.1)	39.5	(38.2)	0.454
Appetite loss	27	41.4	(38.5)	43.9	(35.3)	42.9	(36.4)	0.781
Constipation	30	50.6	(34.1)	17.1	(27.0)	31.0	(34.2)	**<0.001**
Diarrhoea	31	20.7	(28.7)	19.5	(29.8)	20.0	(29.2)	0.868
Financial impact	42	81.6	(36.3)	69.1	(40.4)	74.3	(39.0)	0.180

^a^Numbers as per the item numbers in the questionnaire; ^b^Functional scale scores range from 0 to 100, with a higher score representing a higher level of functioning or QOL; ^c^Symptom scale scores range from 0 to 100, with higher score representing a greater degree of symptom severity

## Discussion

A better understanding of the factors influencing the QOL among patients with advanced illness can facilitate policy development, particularly in resource constrained countries [33]. Herrera and colleagues [34] remind us that PC provision should not be determined by the patient’s geographical location, his/ her disease, or ability to pay, but on needs alone. Other studies [35, 36] further support the contention that needs assessed deliberately among patients provides useful information to plan programmes and interventions that meet the explicit requirements of a local population. The findings of this study, although a relatively small sample, when combined with the findings from the other concurrent studies among family members and health care professionals, are fundamental to informing the development of PC services in Bhutan.

Forty two percent of the patients in the study had advanced cancer. Cancer in Bhutan has increased from 923 new cases in 2013 to 1,824 in 2017. Cancer is identified as a major threat to public health in the developing countries [37]. The remaining 58% were diagnosed with advanced, non-malignant diseases including both communicable and chronic diseases. In 2017, along with 144 cancer deaths, 212 deaths related to heart disease, 166 to alcoholic liver disease and 39 to chronic kidney disease were reported [25]. Substantial numbers of chronic patients also die at home without a cause of death being reported.

When diagnosed with cancer, both the patient and family experience a wide range of needs throughout the illness trajectory, ranging from physical needs to emotional, psychological, social, financial and spiritual needs [38]. The needs of patients with non-malignant conditions are now increasingly identified [39–41]. This research found that people diagnosed with advanced malignant and non-malignant illnesses in Bhutan have poor levels of functioning and varied levels of symptom severity indicating high support needs, consistent with advanced illnesses in other LMICs [33, 42–45]. There was no differences in symptom severity or level of functioning between cancer and non-cancer patients except for constipation (p value < 0.05). This is in contrast to a study of elderly cancer patients in Sweden who had poorer scores, more complaints and subsequently poorer quality of life compared to non-cancer patients [29].

There are no published studies of normative EORTC QLQ-C30 data among Asian PC populations. The existing normative data is most available for general populations and the mean scores for the Global Health/QOL across Australia (68.5, SD 21.5), Columbia, (77.1, SD 18.5), the European countries (66.1, SD 21.7) and Korea (67.7, SD 68.8) provide a picture of quality of life scores for those without advanced illness [46–49]. One study from Ethiopia [33] which focused on people mostly diagnosed with later stage cancer, reported a better mean global health/QOL score than patients with advanced illnesses in Bhutan (Ethiopia 54.6, SD 26.2 compared with Bhutan 47.4, SD 24.1) despite the mean score for pain in cancer patients in the two countries being similar (Ethiopia 55, SD 36.2, Bhutan 55.2, SD 35.7). Symptoms like fatigue and financial difficulties related to the illness are worse among Bhutanese cancer patients (59.4, SD 34.8 and 81.6, SD 36.3, respectively) compared to Ethiopian cancer patients (52.6, SD 36.1, and 67.1, SD 41.2, respectively) and much more than the general population in the aforementioned countries [33, 46–49]. These comparisons highlight the need for a model of PC in Bhutan which considers the social and economic context of patients in addition to their disease and symptom management priorities.

In this study, both cancer and non-cancer patients experienced moderate pain (mean 46.2, SD 37.1). Pain is one of the most common and feared symptoms experienced by patients with advanced illnesses [6], and can significantly influence the psychological and emotional wellbeing and overall QOL of both patients and families [50, 51]. In Bhutan, although oral morphine is available at all levels of healthcare except in BHU Grade II [52], it may be inadequately used as PC is an emerging concept [26]. Where PC is not well developed, several barriers to accessing morphine have been reported, including lack of availability, limited knowledge and opiophobia among physicians, along with a reluctance by patients and families to use it [53, 54]. The need to improve pain management is a priority area for new PC services in Bhutan.

Social problems were far reaching for participants, in addition to their medical condition. Most were married and many had young children. In one case the eldest child was just six years old and in another the youngest was just one. Parenthood is one of the major concerns when diagnosed with an advanced illness [55], however, the needs specific to dying patients and their dependent children are not well met even in the developed countries with comprehensive PC [56–58].

Forty one percent of participants in this study did not go to school and 13% had just primary education— indicating that 54% of the patients had significant problems with literacy. Literacy is an important concern in LMICs given its association with a range of adverse health outcomes [59]. Misinformation and misunderstandings related to PC and hospice services among indigenous populations [60] have emphasized public education as an integral component of PC which provides information, knowledge and skills enabling patients, particularly the marginalized and vulnerable groups, to adapt effectively when faced with the diagnosis of advanced illness [61]. Given the low levels of literacy in Bhutan and a lack of knowledge about PC, even amongst health professionals, education and raising awareness about PC is important for the public in Bhutan. Integrating PC modules in nursing colleges, postgraduate medicine, regular training programs for in-service health professionals combined with PC awareness initiatives for relevant stakeholders including policy makers are some of the important avenues to develop PC education. Use of national television, radio and other social media platforms can be good strategies to create awareness about PC in Bhutan.

Sixty one percent of the patients in this study did not have a reliable job or income, which was reflected in the high levels of financial stress for both cancer and non-cancer patients. Although healthcare in Bhutan, including referral abroad, is funded by the government [62], patients still need to pay for accommodation in private cabins instead of the general hospital ward which is noisy and privacy often compromised; for some medicines that are not included in the essential drug list; and for the services availed from the private diagnostic facilities both within and outside the country [22]. Patients incur expenditure on the repeated travel to and from the referral centres for the treatment and management of their illness. This situation is similar in other countries in the region where rural patients have to travel long distances for treatment and where illness and the time required for treatment further reduces incomes [63, 64]. Moreover, Bhutanese often spend huge amounts of money on rituals, prayers, offerings and other traditional healing practices when confronted with advanced illness because patients and family members strongly believe that diseases are caused by bad, vengeful spirits causing imbalances in the vital elements – bile, phlegm and wind channels, within the body [65, 66]. Traditional healing practices along with modern medicine are considered to provide parallel benefits in cancer management and end-of-life care, especially in developing countries [60, 67–69]. Having traditional medicine integrated into the national health system coupled with well informed and engaged local traditional healers can play a significant role in advancing PC in Bhutan.

Consistent with previous research in both developed and developing countries [28, 42–45, 70, 71], both cancer and non-cancer patients in Bhutan have varied needs throughout their illness that affects their overall QOL—ranging from physical symptom control to psychological, emotional, cognitive and spiritual support, as well as the need for food and financial assistance. Patients also had information needs about diagnosis, treatment options and side effects along with the need for psychological and spiritual support [35, 72].

Palliative care aims to address the broad range of symptoms and needs of patients with advanced illnesses described in our study. When assessing PC needs for a country or a region, it is vital to understand the benefit of such programmes to the local population [73]. The WHO recommends that a PC service should, at a minimum, identify both cancer and non-cancer patients who could benefit from PC; assess, reassess and address their physical, psychological, social, emotional and spiritual distress and determine culturally appropriate goals of care [6]. In rural Africa, PC integrated into disease-modifying therapies and into the routine HIV services have reduced physical pain and improved psychosocial and spiritual wellbeing including socioeconomic assistance for most patients [74, 75]. Concurrent PC among advanced cancer patients is perceived by oncologists to improve the quality of life of patients and families, facilitate better quality of care with less aggressive end-of-life care, and reduce emotional distress [76]. Given that PC is a young concept in Bhutan, these findings are relevant and applicable to the Bhutanese context as education and awareness programs are crucial to introducing PC services.

Patients with advanced illnesses in this study were admitted to the hospital many times since diagnosis, and the majority (59%) spent at least 2 weeks in hospital during each admission. Several studies [10, 18, 77, 78] have found that effective PC services can avoid repeated, often unnecessary, visits to emergency departments at the end-of-life. Palliative care services have also reduced long-term hospital admissions and the referral of patients abroad, thus reducing health care costs. Almost 40% of the health care expenses are known to occur in the last 3 months of life and account for almost 70% of end-of-life expenditures [79]. The government of Bhutan is challenged by the escalating cost of health care [22]. Integrating a public health approach to PC into the existing health care system can help manage the increasing costs.

While the principles of PC remain universal, the WHO and other international organizations reiterate that PC models should be contextually appropriate [1, 8, 9]. Modified to its social, cultural, spiritual and economic context, even resource constrained countries like Bhutan can provide effective PC services as demonstrated by Kerala in India, Arusha in Tanzania, Brasov in Romania, Vietnam and Argentina [1, 15, 80].

### Strengths and limitations

There are several strengths and limitations to this study. Bhutan has varying cultural and belief systems in response to advanced illness, death and dying and post-death rituals. A strength of this study was the inclusion of participants from all regions across the country. Although a sample size of 70 participants is small, the challenges faced to hear those 70 voices were great, and the study would be difficult to repeat for someone not fluent in the local dialects. The small sample size limited the inferential statistics to compare between cancer and non-cancer participants. One of the limitations was the use of only the EORTC-QLQ-30 which resulted in a reduced understanding of other needs such as information, spiritual and sexual needs of the patients. Due to complex practicalities including low literacy, issues with translation and the potential for encountering cultural taboos, we foresaw difficulties in using multiple tools and chose to use just one to maximise participation. This was also the first research conducted with this population in Bhutan. Future studies could consider a bigger sample and include other tools to explore additional needs in such patients.

## Conclusions

The symptom burden experienced by people affected by advanced illnesses demonstrates the need for PC in Bhutan. Bhutan is a small developing nation with a small population and has primary health care and traditional healing systems underpinned by compassion and equity. With a very limited home PC service now initiated, this study of patients’ needs will help inform the development of a broader public health-focused PC model, modified to the Bhutanese social, cultural and economic context, as recommended by the WHO.

## Data Availability

The data that support the findings of this study are available on request from the corresponding author. The data are not publicly available due to privacy and ethical reasons.

## Abbreviations

BHU: Basic Health Unit
EORTC QLQ-C30: European Organisation for Research and Treatment of Cancer Quality of Life Questionnaire
HIV: Human Immunodeficiency Virus
JDWNRH: Jigme Dorji Wangchuck National Referral Hospital
LMIC: Low and Middle Income Countries
PC: Palliative Care
QOL: Quality of Life
SAS: Statistical Analysis System
SD: Standard Deviation
WHO: World Health organization
